# Plasmid co-infection: linking biological mechanisms to ecological and evolutionary dynamics

**DOI:** 10.1098/rstb.2020.0478

**Published:** 2022-01-17

**Authors:** Claudia Igler, Jana S. Huisman, Berit Siedentop, Sebastian Bonhoeffer, Sonja Lehtinen

**Affiliations:** ^1^ Institute of Integrative Biology, Department of Environmental Systems Science, ETH Zürich, Zurich, Switzerland; ^2^ Swiss Institute of Bioinformatics, Lausanne, Switzerland

**Keywords:** plasmids, mathematical modelling, frequency-dependent selection, ecological and evolutionary dynamics, plasmid co-infection

## Abstract

As infectious agents of bacteria and vehicles of horizontal gene transfer, plasmids play a key role in bacterial ecology and evolution. Plasmid dynamics are shaped not only by plasmid–host interactions but also by ecological interactions between plasmid variants. These interactions are complex: plasmids can co-infect the same cell and the consequences for the co-resident plasmid can be either beneficial or detrimental. Many of the biological processes that govern plasmid co-infection—from systems that exclude infection by other plasmids to interactions in the regulation of plasmid copy number—are well characterized at a mechanistic level. Modelling plays a central role in translating such mechanistic insights into predictions about plasmid dynamics and the impact of these dynamics on bacterial evolution. Theoretical work in evolutionary epidemiology has shown that formulating models of co-infection is not trivial, as some modelling choices can introduce unintended ecological assumptions. Here, we review how the biological processes that govern co-infection can be represented in a mathematical model, discuss potential modelling pitfalls, and analyse this model to provide general insights into how co-infection impacts ecological and evolutionary outcomes. In particular, we demonstrate how beneficial and detrimental effects of co-infection give rise to frequency-dependent selection on plasmid variants.

This article is part of the theme issue ‘The secret lives of microbial mobile genetic elements’.

## 1. Introduction

Plasmids are mobile genetic elements of bacteria that play a fundamental role in a variety of areas, including bacterial evolution [1,2], clinical infections [3,4] and biotechnology [5,6]. Naturally occurring plasmids exhibit considerable diversity, both in the genes necessary for plasmid replication and spread (plasmid backbone) [7–10], and ‘cargo’ genes, which do not directly impact the plasmid but affect the fitness of the host cell. Such cargo genes can encode traits including antibiotic resistance [11,12], heavy metal tolerance [13], virulence [14] and toxins for inter-strain competition [15].

The ecological interactions which shape this diversity are complex: plasmids compete for a limited resource—host cells to infect—but host cells often carry more than one type of plasmid (co-infection) [16–18]. The interactions between co-resident plasmids play a major role in shaping plasmid ecology and evolution. On the one hand, competitive within-cell interactions exert a strong selective pressure on the plasmid backbone, for example by driving the diversification of plasmid replication machinery [19] or the development of systems aimed at hindering co-resident plasmids [8,10]. Particularly, many plasmids carry systems that prevent co-infection with closely related plasmids, indicating the importance of reducing intra-cellular competition [7]. On the other hand, within-host interactions can also be beneficial for one or both of the co-resident plasmids. This benefit can arise from increased horizontal transmission, for example through increased conjugation rates from co-infected cells to recipient cells [20]; or from increased vertical transmission (i.e. plasmid inheritance to daughter cells), for example through a reduced metabolic burden of a particular plasmid variant when carried with another plasmid variant rather than alone [18,21]. Not all plasmids are conjugative (i.e. capable of independent horizontal transfer), but some non-conjugative plasmids can hitchhike along with the conjugation apparatus of co-infecting plasmids [22,23], making them ‘mobilizable’. Overall, within-host interactions crucially shape the fitness landscape plasmids exist in, and thus their population dynamics and diversity.

The (known) biological processes shaping plasmid co-infection have been studied in considerable mechanistic detail [19,24–27]. Given the complex interactions between these processes and the difficulties in scaling experimental systems to capture many genetic and environmental conditions, mathematical modelling plays a central role in translating mechanistic insights into predictions about plasmid dynamics and diversity in nature. For example, models of co-infection have provided insights into the conditions for coexistence of conjugative plasmids [28–31]; the maintenance of non-conjugative plasmids [32,33]; factors influencing gene mobility between plasmids [34]; and the evolution of specific traits such as surface exclusion [28] and toxin-antitoxin systems [35].

Existing models have proved useful in understanding specific aspects of co-infection, but here we develop a more general framework relating co-infection processes to ecological and evolutionary outcomes. This approach is particularly important because constructing appropriate models of co-infection is not trivial: theoretical work on co-infection between disease strains has shown that seemingly innocuous modelling choices can introduce unintended ecological differences between strains, with considerable impact on model outcomes [36–38]. In particular, model structures easily introduce mechanisms which unintentionally promote strain diversity (coexistence for free) [36]. Models of plasmid conjugation are structurally similar to these epidemiological models of infectious disease transmission, making these concerns about implicit modelling assumptions also relevant for plasmid co-infection.

Our aim is to develop a synthesis of how the biological processes governing co-infection influence the outcomes of plasmid competition. We begin by constructing a general model of co-infection by abstracting many of the processes involved, which allows for flexibility in implementing the underlying biological mechanisms. These different possibilities of implementation are discussed in the context of a literature review on the relevant features of plasmid co-infection. We proceed by giving an intuition of how various co-infection parameters affect bacterial population diversity and by developing a general relationship between co-infection and evolutionary outcomes. Finally, we summarize the main findings of our synthesis and give an outlook on future experimental and theoretical explorations arising from it.

## 2. A model of plasmid co-infection

**Figure 1.**
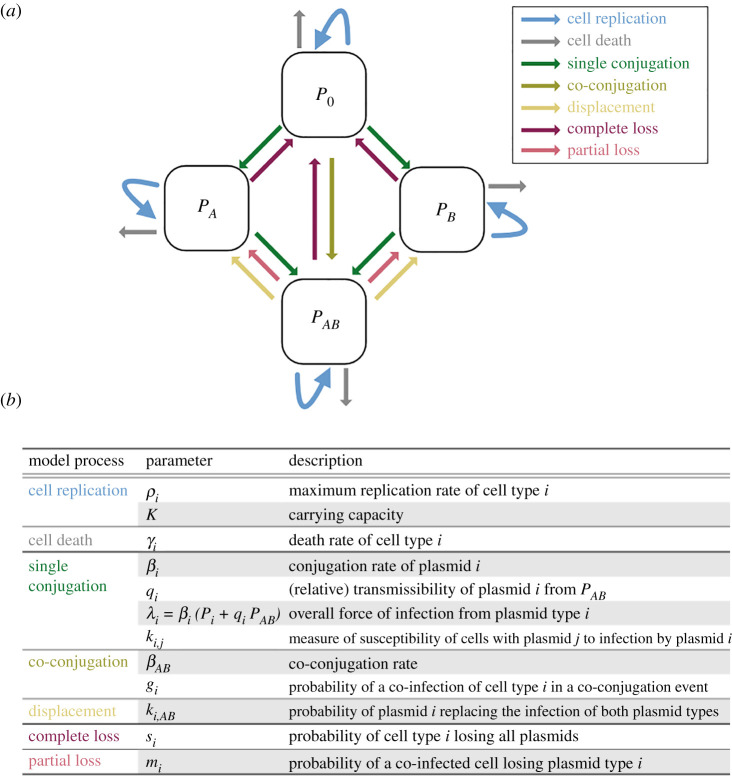
Visualization of the modelled plasmid co-infection processes and the corresponding parameters. (*a*) Schematic diagram of the co-infection model given by equations (2.1). *P*_0_ denotes plasmid-free cells, *P*_*A*_ and *P*_*B*_ are bacterial cells infected with plasmid variant *A* or *B*, respectively, and *P*_*AB*_ are cells co-infected with *A* and *B*. Arrows indicate the transition of cells between states. (*b*) Co-infection processes incorporated in the model, listed with their associated parameters and parameter descriptions.

We begin by developing a model of the population dynamics of two plasmid variants, *A* and *B*, (co-)infecting a bacterial population. This model tracks the density of cell populations in terms of their infection status: no plasmid (*P*_0_), plasmid *A* (*P*_*A*_), plasmid *B* (*P*_*B*_) or co-infected with both plasmids (*P*_*AB*_). We are specifically interested in the effects of vertical and horizontal transmission. Hence, our exploration focuses on conjugative plasmids, but the same model structure would also be appropriate for a pair of plasmids where one is conjugative and one mobilizable. The model captures the following fundamental steps in the life-cycle of conjugative plasmids. Plasmids reside within bacterial cells at a copy number determined by the plasmid backbone, which can range from 1–10 [39] to up to 200 [40] copies per cell. (Note that we do not explicitly model copy number.) Resident plasmids can be transmitted either vertically via host cell replication, or horizontally via conjugation. Vertical transmission requires plasmid replication and partitioning within the cell such that both daughter cells inherit at least one plasmid copy. Conjugation requires expression of transfer genes and close contact between a recipient and a donor cell, allowing transfer of a plasmid copy. The recipient may already carry another plasmid, resulting in co-infection. Co-residence of two (or more) plasmid variants can impact each of these processes and even prevent some from taking place at all. The detailed biological mechanisms will be discussed in §3. First, we develop a more conceptual intuition of these processes through their realization in a mathematical model (figure 1; more details on model structure are given in the electronic supplementary material, text 2 and table S1).

### (a) Bacterial population size

We model changes in the host cell density in two components: (i) a density-dependent *replication* rate *ρ*_*i*_(1 − (*T*/*K*)), with *ρ*_*i*_ representing the maximum replication rate, *i* the cell type (0, *A*, *B* or *AB*), *T* the total cell density (*T* = *P*_0_ + *P*_*A*_ + *P*_*B*_ + *P*_*AB*_) and *K* the carrying capacity; and (ii) a density-independent *death* rate *γ*_*i*_. Plasmid costs and benefits can be captured in both *ρ*_*i*_ and *γ*_*i*_, for each cell type individually.

### (b) Plasmid conjugation

#### (i) Single conjugation

Plasmids conjugate in a manner dependent on host cell density, at rate *β*_*i*_, where *i* indicates plasmid variant *A* or *B*. The relative transmissibility of plasmid *i* from co-infected cells (*P*_*AB*_), is given by *q*_*i*_. Thus, the overall force of infection from plasmid variant *i* is *λ*_*i*_ = *β*_*i*_(*P*_*i*_ + *q*_*i*_ *P*_*AB*_).

If the recipient cell is already (singly) infected with plasmid variant *j*, further infection with plasmid variant *i* is possible, and leads to co-infection. The susceptibility of cells with (only) plasmid *j* to infection by plasmid *i*, relative to cells with no plasmid, is given by *k*_*i*,*j*_.

If the recipient is already co-infected, further infection with either variant can theoretically lead to *displacement* of the co-resident variant, and a return to a singly infected state (known as ‘knock-out’ in the epidemiological modelling literature [36]). The probability of plasmid *i* displacing plasmid *j* from a co-infected cell upon infection is given by *k*_*i*,*AB*_. This parameter subsumes two biological processes: infection with plasmid *i*, and loss of the other plasmid variant already present in the co-infected cell. These processes are related because the increased within-host copy number of variant *i* makes loss of variant *j* more likely (see §3; electronic supplementary material, text 3.3).

#### (ii) Co-conjugation

If co-infected cells can also transmit both plasmids simultaneously (co-transfer), co-conjugation from *P*_*AB*_ occurs at rate *β*_*AB*_. Hence, the overall infectiousness of co-infected cells is given by *q*_*A*_*β*_*A*_ + *q*_*B*_*β*_*B*_ + *β*_*AB*_. If the recipient carries no plasmid (*P*_0_), it transitions directly to the *P*_*AB*_ state. If the recipient is singly infected, e.g. *P*_*A*_, co-conjugation leads to co-infection with probability *g*_*A*_.

### (c) Plasmid segregation loss

#### (i) Complete loss

Cells can lose (single or double) plasmid carriage completely during cell division (*s*_*i*_).

#### (ii) Partial loss

Co-infected cells can revert to being singly infected if they lose only one plasmid variant. This occurs with probability *m*_*i*_ (with the constraint *m*_*A*_ + *m*_*B*_ ≤ 1). Note that, depending on the specific mechanism of plasmid loss in co-infected cells, *s*_*i*_ and *m*_*i*_ may not be independent, which can be captured by constraining their relationship.

These processes are captured by the following equations:

2.1 $$\begin{array}{rl} & \frac{dP_{0}}{dt}=P_{0}\left[\rho_{0}\left(1-\frac{T}{K}\right)-\gamma_{0}-\lambda_{A}-\lambda_{B}-\beta_{AB}P_{AB}\right]\\ & \;+\left(1-\frac{T}{K}\right)[\rho_{A}s_{A}P_{A}+\rho_{B}s_{B}P_{B}+\rho_{AB}s_{AB}P_{AB}]\\ & \frac{dP_{A}}{dt}=P_{A}\left[\rho_{A}(1-s_{A})\left(1-\frac{T}{K}\right)-\gamma_{A}-k_{B,A}(\lambda_{B}\;+\;g_{A}\beta_{AB}P_{AB})\right]\\ & \;+\lambda_{A}(P_{0}\;+\;k_{A,AB}P_{AB})\;+\;m_{B}\rho_{AB}(1-s_{AB})\left(1-\frac{T}{K}\right)P_{AB}\\ & \frac{dP_{B}}{dt}=P_{B}\left[\rho_{B}(1-s_{B})\left(1-\frac{T}{K}\right)-\gamma_{B}-k_{A,B}(\lambda_{A}\;+\;g_{B}\beta_{AB}P_{AB})\right]\\ & \;+\lambda_{B}(P_{0}\;+\;k_{B,AB}P_{AB})\;+\;m_{A}\rho_{AB}(1-s_{AB})(1-\frac{T}{K})P_{AB}\\ & \frac{dP_{AB}}{dt}=P_{AB}[\rho_{AB}(1-s_{AB})(1-m_{A}-m_{B})\left(1-\frac{T}{K}\right)\\ & \;+\;\beta_{AB}(P_{0}+g_{A}k_{B,A}P_{A}+g_{B}k_{A,B}P_{B})\\ & \;-\;\gamma_{AB}-k_{A,AB}\lambda_{A}-k_{B,AB}\lambda_{B}]\;+\;k_{B,A}\lambda_{B}P_{A}+k_{A,B}\lambda_{A}P_{B}. \end{array}$$

## 3. Model parameters: biological mechanisms

Having introduced the basic processes involved in plasmid co-infection, we will briefly portray the underlying complexity of biological mechanisms and how these can be incorporated into our model structure.

### (a) Mechanisms affecting vertical transmission

#### (i) Effect on host cell fitness

The effect of plasmids on the fitness of their host cells can be positive or negative. Hence, co-infection can impact the vertical transmission of co-resident plasmids through effects on host cell replication or death (*ρ*_*AB*_, *γ*_*AB*_). Importantly, these effects may be different than expected from the effects of each plasmid individually (epistasis). For example, there is empirical evidence of positive epistasis (i.e. reduced fitness costs) between co-infecting plasmids [18,21], which could stem from down-regulation of the conjugation machinery [41] (see below) and/or a decrease in the number of individual plasmid copies per cell [42]. Epistatic effects could also arise from interactions between plasmid cargo genes, e.g. diminishing returns epistasis, whereby the additional benefit of a cargo gene is lower in a fitter background (for instance with resistance genes for the same antibiotic on two different plasmids) [43].

#### (ii) Plasmid replication and partitioning

The most important steps in faithful vertical plasmid transmission are plasmid replication and (for some plasmids) partitioning, which positions plasmid copies within the cell to ensure inheritance to both daughter cells. When co-infecting plasmid variants share the same replication and/or partitioning regulation, either variant is more likely to be lost during cell division. This leads to an inability of plasmid variants to coexist stably in the same cell lineage, which is used to define plasmid incompatibility [19]. However, as this definition is based on a phenotype, ‘incompatibility’ can also arise from other within-host interactions [44]. The speed at which incompatibility eliminates within-cell coexistence depends on the cause of incompatibility (see below) and the plasmid gene content (e.g. toxin-antitoxin systems): estimates of segregation loss rates for identical co-resident plasmids include 1–15% [45] and 16–22% [46] per replication (electronic supplementary material, table S2).

#### (iii) Replication systems

Plasmid replication, and hence plasmid copy number in the cell, is tightly regulated to minimize the cost to the host—while still guaranteeing stable vertical transmission. Generally, the distribution around the target copy number within each cell is a narrow Gaussian [47], although recent evidence shows that the standard deviation can be of the order of the mean copy number [48]. Replication control is based on feedback from the plasmid copy number in the cell (down-regulation at high copy numbers) [19]. Hence, incompatibility arises from the inability of plasmids to differentiate between their own and the co-resident’s copy number and correct for deviations from the target number [19]. Two plasmid variants sharing replication determinants will establish the same overall copy number as they would individually, but with a mixed plasmid pool. Random sampling from this pool for replication leads to heterogeneity in the within-host frequencies of the two plasmid variants [19]. In the absence of other effects (including conjugation), genetic drift will lead to eventual loss of all copies of one plasmid variant from the cell lineage (electronic supplementary material, table S2).

#### (iv) Partitioning systems

To ensure stable inheritance to both daughter cells, sibling plasmids have to be separated into the two cell halves after replication. This is especially important for low copy number plasmids, which are known to use partitioning systems for this purpose. However, non-random positioning has also been found for high copy number plasmids [49], which is beneficial if heterogeneity in copy number can indeed be large [48].

Partitioning systems generally consist of three (plasmid-encoded) components: a centromere-like DNA site and two proteins, an NTPase (energy production and movement) and a centromere-binding protein (plasmid tethering) [50]. The incompatibility mechanism is determined by the affected component and can lead, for example, to random partitioning or centromere-binding protein sequestration [51]. The variation that is found in centromere-like DNA sites alone indicates selection pressure for distinct partitioning systems [51]. Notably, some plasmids harbour multiple partitioning systems, which can increase their stability compared to either system alone [52]. Similarly, the presence of multiple replication systems on one plasmid has been reported [16,53]. This can provide the benefit of a broad host range [53], or of incompatibility avoidance [16].

The influence of partitioning and replication systems on plasmid co-infection differs depending on their relatedness (electronic supplementary material, figure S6):

— identical replication systems: complete and partial segregation loss are symmetrical (*s*_*AB*_ = *s*_*A*_ = *s*_*B*_, *m*_*A*_ = *m*_*B*_). Partial segregation loss is more frequent than for compatible plasmids (electronic supplementary material, table S2), especially if partitioning is also incompatible [54];
— related replication systems: partial segregation probabilities can be either symmetric or favour the plasmid that is less sensitive to the incompatibility determinant. Higher stability could also be related to a difference in copy number, as higher numbers increase the chance of being selected as a replication template [19]; and
— compatible replication systems: incompatibility can still arise via partitioning systems only. Again, this can lead to symmetric or asymmetric segregation loss for co-resident plasmids. Interestingly, for low copy number plasmids with partitioning incompatibility, loss rates can be even higher (fourfold to fivefold) than those arising from random partitioning [55].

Replication and partitioning also influence susceptibility of plasmid carriers to further infection, i.e. to co-infection, *k*_*i*,*j*_, for singly infected cells and displacement, *k*_*i*,*AB*_, for co-infected cells. In the case of co-infection, a newly co-infecting plasmid variant will have a low copy number compared to the established variant, thus making it more likely to be lost during the first rounds of cell replication, if the previously established plasmid is incompatible. In the case of displacement, further infection with one of the variants will increase the within-cell frequency of this variant. If the co-resident variants are incompatible, this will increase the probability that the lower frequency variant will not be selected for replication or will be lost during cell division. Finally, if segregation loss of one of the incompatible plasmid variants is very rapid, co-infection becomes negligible and need not be modelled at all. However, as discussed above, current estimates suggests that segregation loss is slow (1–22% probability of one co-resident variant being lost per generation–see the electronic supplementary material, table S2).

Replication and partitioning systems impact a number of other model parameters indirectly, since they lead to a lower copy number of each plasmid variant in the co-infected cell. This can decrease the probability of successful conjugation (*q*_*i*_) [56] and plasmid cost (*ρ*_*AB*_, *γ*_*AB*_), compared to co-infection with compatible plasmids.

#### (v) Toxin-antitoxin systems

Toxin-antitoxin (TA) systems on plasmids are usually seen as addiction modules that select against plasmid-free cells through ‘post-segregational killing’ [57]: after plasmid loss, neither toxin nor antitoxin is produced any longer, but the more stable toxin persists (without antitoxin) in the cell and interferes with essential cellular processes like replication, translation and cell-wall synthesis [58]. However, toxin inhibition of cell metabolism seems generally reversible (e.g. the F plasmid toxin inhibits cell division only until completion of plasmid replication [59]), with cell killing only being observed in over-expression experiments [60]. This suggests that TA systems act both to reduce competition from cells that have lost the plasmid and to increase faithful inheritance [59].

While TA systems have been found to promote plasmid maintenance, they seem to be (up to a 100-fold) less efficient than partitioning systems [58] (electronic supplementary material, table S2). Their overall stabilization effect varies considerably (2.5-100-fold) and is dependent on the host strain [61] (electronic supplementary material, table S2). The impact of TA systems during co-infection could be greater, as loss of the TA-carrying plasmid will slow down vertical and horizontal transmission of the non-TA-carrying plasmid [8].

The influence of plasmid TA systems can be modelled in various ways (table 1):

— if TA systems kill the plasmid-free host, segregation loss leads to cell death instead of transition to the plasmid-free state. This can be modelled by introducing a (1 − *x*) modifier to the complete segregation loss term (*s*_*i*_) in the equation for *P*_0_ (only): a proportion *x* of cells that lose the plasmid die. For co-infection with a TA-carrying (*A*) and non-TA-carrying (*B*) plasmid, partial segregation loss (*m*_*A*_) and displacement (*k*_*B*,*AB*_) can be similarly modified in the equation for *P*_*B*_ to capture cell death following the loss of plasmid *A*; and
— if TA systems inhibit cell division until plasmid replication is completed, the increased vertical stability can be modelled by decreasing complete (*s*_*i*_) and partial segregation loss (*m*_*i*_), at the cost of a lower replication rate (*ρ*_*i*_). The increased division time may also increase vertical stability (i.e. decrease *m*_*i*_) of a co-resident plasmid. The decreased competitiveness of cells that have lost the TA-carrying plasmid would be most accurately represented by introducing additional states to capture the temporary reduction in post-segregational replication rate. To avoid the introduction of additional states, the effect may be approximated by modelling the decreased net growth rate through post-segregational death (i.e. as above).

**Table 1.** Summary of biological processes relating to co-infection and their relationship to model parameters.

biological process	model parameter	mechanism
replication	*m*_*i*_, *s*_*AB*_	crosstalk in replication regulation
replication, partitioning	*q*_*i*_	decreased number of plasmid copies (gene dosage)
partitioning, segregation	*m*_*i*_, *s*_*AB*_	crosstalk in partitioning components
segregation	*s*_*i*_	stochasticity in plasmid inheritance (single infection)
	*s*_*i*_(1 − *x*), *m*_*i*_(1 − *x*)	TA-induced stabilization (single and double infection)
cell growth	*ρ*_*i*_, *γ*_*i*_	toxin inhibition of cell metabolism
	*ρ*_*AB*_, *γ*_*AB*_	epistasis in plasmid costs
	*ρ*_*AB*_, *γ*_*AB*_	fertility inhibition systems
conjugation, donor	*β*_*AB*_, *q*_*i*_	fertility inhibition systems
	e.g. *β*_*AB*_ = min(*β*_*A*_, *β*_*B*_)	synchronized de-repression of conjugation machineries (co-transfer)
	*q*_*i*_, *β*_*AB*_	co-integrates
conjugation, recipient	*k*_*i*,*j*_, *k*_*i*,*AB*_	exclusion systems (*cis*- or *trans*-acting)
	*k*_*i*,*j*_, *k*_*i*,*AB*_	high probability of loss immediately after co-infection owing to replication (partitioning) incompatibility
	*k*_*i*,*AB*_	TA-induced death

### (b) Mechanisms affecting horizontal transmission

#### (i) Conjugation from co-infected cells

A key characteristic of conjugative plasmids is their ability to transmit themselves horizontally to neighbouring cells, which requires the expression of transfer genes from the plasmid, and close proximity between the recipient and donor cell.

To reduce the burden on the host, the conjugation machinery is generally downregulated (repressed) and not active at all times [62]. Plasmids typically carry fertility inhibition systems, which inhibit conjugation, either as an auto-regulatory mechanism (F plasmids), or to inhibit transfer of unrelated, co-resident plasmids [10,63] (electronic supplementary material, table S2). Activation is also influenced by diverse factors such as host cell physiology, the availability of recipients, or stress factors like antibiotics [64–66]. Such external activation signals can de-repress both co-infecting plasmids, increasing the chance of simultaneous transfer [67].

Co-infecting plasmids can affect each other’s individual conjugation rates (*q*_*A*_, *q*_*B*_), as well as transfer simultaneously during a single mating event (co-transfer; *β*_*AB*_). Effects on individual conjugation rates during co-infection seem common (63% of tested plasmid pairs), although typically only one plasmid is influenced (53% of plasmid pairs) [20]. In this case, a reduction in conjugation rate was more commonly observed (30%) than an increase (23%) [20].

Co-transfer of plasmids can occur through the same type IV secretion system (T4SS), or by expression of several systems simultaneously. Mobilizable plasmids can ‘hitch-hike’ along with the T4SS of a conjugative plasmid, if they encode compatible transfer determinants [22,23]. Transfer via the same T4SS can also occur with plasmid co-integrates [68], which arise through fusion of plasmid variants.

In the case of multiple co-resident, conjugative plasmids, simultaneous expression of the T4SS could stabilize the mating pair, thus allowing efficient co-transfer [20]. However, determination of the true rate of conjugative co-transfer is difficult as ‘simply’ counting the number of recipients that received both plasmids does not allow one to distinguish whether a single or two subsequent mating events took place. This may explain the variation in empirical co-transfer reports, showing frequent co-transfer in a system with large and small plasmids [69], and in an engineered system with conjugative plasmids [67], but little in another system with conjugative plasmids from natural isolates [70].

The effect of co-infection on conjugation can be modelled in the following ways (table 1):

— fertility inhibition systems decrease the individual and co-conjugation rate (*q*_*i*_, *β*_*AB*_) of co-resident plasmids, resulting in up to 10 000-fold lower conjugation rates [63]. Lower conjugation rates might in turn decrease the plasmid burden on the host cell (*γ*_*AB*_, *ρ*_*AB*_) [41];
— co-transfer rates of co-resident plasmids are largely unknown, but have been proposed to occur at the rate set by the lower conjugation frequency (*β*_*AB*_ = min(*β*_*A*_, *β*_*B*_)) [67]; and
— co-integrates, i.e. fused plasmid variants, can increase (higher probability of expressing at least one conjugation machinery) [71] or decrease (lower mating pair stability) the rate of co-conjugation (*β*_*AB*_), and hence the total conjugation frequency of individual plasmids ($q_{i}\beta_{i}+\beta_{AB}≶\beta_{i}$). Note that our model only captures this process if co-integrates are resolved again after transfer.

#### (ii) *Cis*-acting prevention of co-infection

Conjugative plasmids carry genes with which they can prevent co-infection by plasmids from the same exclusion class (i.e. *cis*-acting) [7]. This serves to reduce: (i) within-host competition between plasmids, (ii) the metabolic burden of conjugation on donor cells, and (iii) recipient death owing to excessive DNA transfer (lethal zygosis) [7]. There are two types of exclusion systems: surface exclusion (SFX), which inhibits the ability to form stable mating pairs, and entry exclusion (EEX), which inhibits DNA transfer across the mating channel. While the latter is found in nearly all conjugative plasmids, only plasmids with pili that firmly attach to the recipient cell code for surface exclusion [7,63].

For F plasmids, entry exclusion was found to be around 10 times more effective than surface exclusion [9,25,26,72]. Together, these systems can generate differences in plasmid transfer between 100-10 000-fold (individually, 200- and 20-fold for EEX and SFX, respectively) [25,26,72]. Similarly, 10-10 000-fold reductions in transfer have been observed for EEX with plasmids of other incompatibility groups [7,73]. The width of this range is probably owing to differences in plasmid copy number, as exclusion was found to be gene dosage dependent [7,72,73].

Despite the ubiquity of exclusion systems, in practice their impact remains unclear. First, there is substantial genetic diversity between SFX and EEX genes, and how this translates into the exclusion phenotype is not well understood. Within the group of F-like plasmids, at least four different surface exclusion groups were identified [74], where specificity was determined only by a difference of five amino acids [75]. The EEX gene is less conserved than the SFX gene: homologous EEX genes were found in only 30% of 256 F-plasmids [76]. Second, certain broad host range plasmids exhibit ‘retrotransfer’, whereby the plasmid is transferred into a recipient, ‘captures’ chromosomal genes or a mobilizable plasmid from that recipient, and is then able to transfer back into the original plasmid-carrying host [77]. Third, little is known about the effect co-resident plasmids have on exclusion. In one experiment, a donor with two plasmids carrying different SFX systems managed to infect a recipient with either one of these plasmids [74]. Fourth, plasmids can bypass exclusion systems by being taken up via a different route (e.g. via transformation, transduction or vessication) [1]. Lastly, exclusion is not active when recipients are in stationary phase [74,78], allowing infection by plasmids from metabolically active donors, or by plasmids that can conjugate in stationary phase [64].

In our model, the parameters describing co-infection susceptibility *k*_*i*,*j*_ and displacement *k*_*i*,*AB*_ can account for exclusion (table 1):

— if exclusion systems are highly effective, modelling co-infection is only relevant for plasmids of different exclusion groups. Co-infected cells would exclude further entry and displacement by either plasmid type (*k*_*i*,*AB*_ = 0); and
— with less effective exclusion systems, cells may be infected by plasmids of the same exclusion group. Co-infected cells can therefore be further infected with either plasmid variant, which can lead to displacement (*k*_*i*,*AB*_ > 0) of one variant. If co-infecting plasmids are of the same exclusion and incompatibility groups, the relationship between *k*_*i*,*j*_ and *k*_*i*,*AB*_ needs to be constrained to avoid introducing unintended ecological differences between the plasmid variants [36]. This is discussed in depth in the electronic supplementary material, text S3.

#### (iii) *Trans*-acting prevention of co-infection

Plasmids can also affect the entry and establishment of other variants into a cell ‘in *trans*’, for example via restriction modification (RM) systems and CRISPR (clustered regularly interspaced short palindromic repeats)-Cas (CRISPR associated systems) [63,79,80], or by affecting cell envelope composition [81].

Restriction-modification systems consist of two functional parts: one cleaves DNA at specific restriction sites, and the other continuously modifies (methylates) these sites to avoid cleavage. This serves primarily as defence against incoming, non-methylated DNA, which will be cleaved upon entry. DNA within the same cell is protected, as long as methylation is actively maintained. If an RM system is lost and the methylation wears off, the remaining restriction endonucleases can kill the cell (i.e. akin to post-segregational killing by TA systems). RM systems are typically located on the chromosome, but are also found in approximately 20% of mobilizable and conjugative plasmids [82]. A resident RM-carrying plasmid can exclude incoming plasmids with non-methylated restriction sites [83,84]. In the case of co-infecting, incompatible plasmids, post-segregational killing will also introduce an advantage for the plasmid with the RM system [44,85]. On the other hand, co-infecting compatible plasmids with RM systems may improve each others conjugation success, by modifying restriction sites that would otherwise be targeted upon entry into a recipient with an RM system.

CRISPR-Cas are systems used by bacteria to defend against mobile genetic elements (MGEs). They typically consist of a ‘library’ of DNA fragments from past MGE infections (called ‘spacers’), and a system that cleaves any of those sequences once they are found in the cell [80]. CRISPR arrays, isolated cas genes, and entire CRISPR-Cas have been found on plasmids [79,80,86]. Generally, CRISPR spacers on plasmids exhibit a strong bias towards other plasmids [86]. CRISPR Type IV systems are even almost exclusively found on plasmids and specifically target the transfer genes of conjugative plasmids [79]. Such systems can keep competing plasmids from establishing in the cell. Importantly, plasmid and chromosomal CRISPR-Cas can acquire immunity to plasmids they were previously (co-)infected with, thus shaping future infection dynamics.

*Trans*-acting exclusion systems can be implemented as follows:

— they lower the chance of successful plasmid transfer to recipients already carrying a plasmid (i.e. *k*_*i*,*j*_ < 1); and
— post-segregational host killing owing to plasmid-borne RM systems can be modelled similar to a TA system (see above).

## 4. Model application

In this section, we examine the influence of modelled co-infection processes on plasmid diversity. Our aim is to provide qualitative conceptual insights; the scale of our parameters is therefore arbitrary (electronic supplementary material, table S1). We begin by considering two ecologically indistinguishable plasmid variants. This means that parameters values are identical for both variants (*β*_*A*_ = *β*_*B*_ = *β*_*AB*_, *k*_*A*,*B*_ = *k*_*B*,*A*_, etc.; electronic supplementary material, table S1). Furthermore, by fulfilling a specific set of requirements (see the electronic supplementary material, text 3), we ensure that the model structure does not implicitly introduce an ecological difference between the variants (structural neutrality) [36].

### (a) Influence of model parameters on co-infection

**Figure 2.**
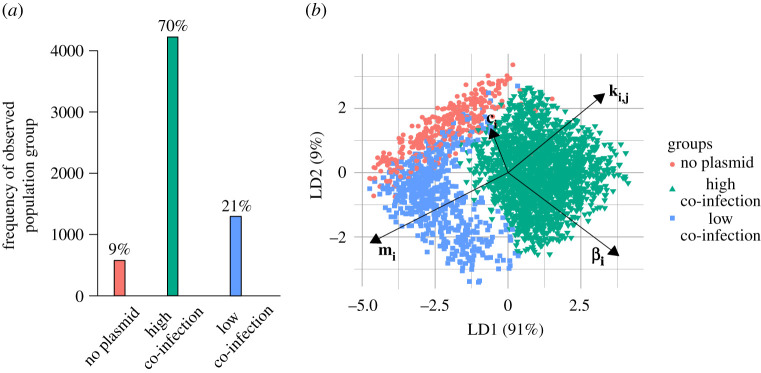
Parameter space exploration using linear discriminant analysis (LDA). (*a*) Probability of each class over all simulation outcomes. Frequencies of each class at the end of 500 time steps - ‘no plasmid’ (red), ‘high co-infection’ (green) or ‘low co-infection’ (blue)—are given for 6100 parameter sets randomly sampled over [0, 0.5] for *m*_*i*_ and [0, 1] for *k*_*i*,*j*_ (= 2*k*_*i*,*AB*_), *β*_*i*_ and *c*_*i*_. (*b*) LDA using the three classes shown in (*a*) (same colour scheme). Arrows show the magnitude and direction of the parameters varied (e.g. the shorter arrow of *c*_*i*_ indicates lower significance of this parameter in class separation, whereas *m*_*i*_ and *k*_*i*,*j*_ (*k*_*i*,*AB*_) are most important in separating high from low co-infection areas).

We begin by providing an intuition for the link between various model parameters and plasmid co-infection states by exploring the parameter space for plasmid conjugation (*β*_*i*_), infection susceptibility (*k*_*i*,*j*_), partial segregation loss (*m*_*i*_) and plasmid cost (*c*_*i*_, defined here as a decrease in replication rate owing to plasmid carriage: *ρ*_*i*_ = *ρ*_0_(1 − *c*_*i*_)). We randomly sample these parameters 6100 times (electronic supplementary material, table S1) and classify the population output at steady state (i.e. no further change when increasing the simulation time) into the following outcomes, as given by thresholds on population frequencies (see the electronic supplementary material, text 1.2): ‘no plasmid’ (*P*_0_), ‘high co-infection’ (*P*_*AB*_) or ‘low co-infection’ (*P*_*A*_ and *P*_*B*_). The frequencies of each class over the whole dataset show by far the highest prevalence of high co-infection (figure 2*a*).

Next, we identify the impact of each parameter on population dynamics using linear discriminant analysis (LDA). Briefly, LDA maximally separates the parameter regions, which tend to result in the different classes defined above [87]. We find that the most significant factors separating the two co-infection classes are susceptibility and partial segregation loss (as shown by the parameter arrows in figure 2*b*), with increases in *k*_*i*,*j*_ leading to more co-infections and increases in *m*_*i*_ resulting in more single infections. The ‘no plasmid’ class is separated from the other two by low conjugation rates and high costs. While higher conjugation rates lead to more plasmid carriage in general, the direction of the arrow indicates that co-infections are relatively more increased. Notably, the magnitude of plasmid cost has the least influence on population outcome among these parameters, though this result may be sensitive to the overall parametrization. On the whole, the co-infection parameters described here affect population outcomes in an intuitive and biologically meaningful manner.

### (b) Co-infection affects evolutionary outcomes through frequency-dependent selection

**Figure 3.**
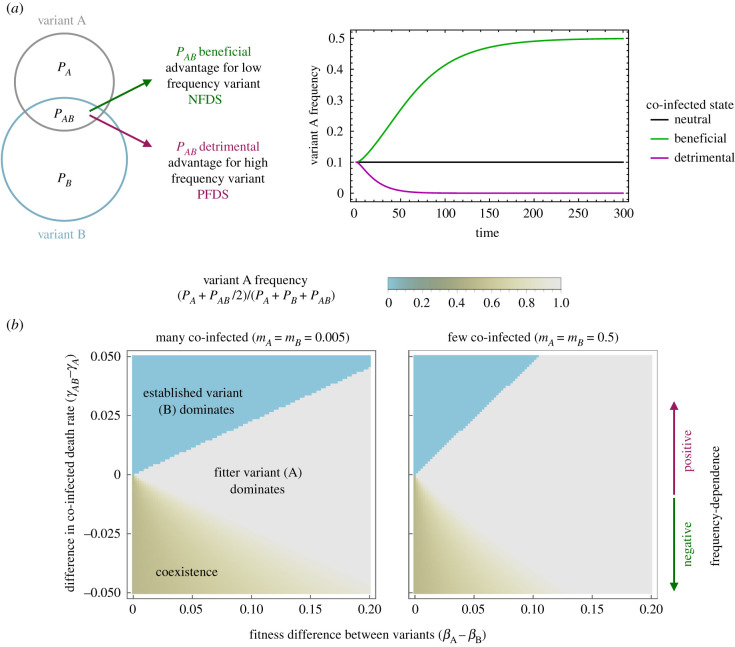
Co-infection affects evolutionary outcomes through frequency-dependent selection. (*a*) The effect of the co-infected state on the outcome of competition between two plasmid variants with identical properties. When the co-infected state is neither beneficial nor detrimental, there is no frequency-dependent selection and the plasmid variants remain at their initial frequencies. A co-infection related advantage for both variants introduces negative frequency-dependent selection (NFDS), which equalizes variant frequencies and leads to coexistence. A co-infection related disadvantage introduces positive frequency-dependent selection (PFDS), which leads to the exclusion of the variant with a lower initial frequency. (*b*) The effect of frequency-dependent selection on evolutionary outcomes in presence of fitness differences between otherwise identical plasmid variants. The figures show the equilibrium frequency of a variant with a fitness advantage but with low initial frequency (*P*_*A*_ = 0.001 and *P*_*B*_ = 1 at *t* = 0). The colour indicates the equilibrium frequency of variant A (here defined as *P*_*A*_ + *P*_*AB*_/2 at *t* = 300 000). The *x*-axis captures the extent of the fitness difference. Here, we implement this as a difference in conjugation rate (*β*_*i*_); fitness differences between variants could also arise from differences in e.g. segregation rate or cost. The *y*-axis captures the strength and direction of the frequency-dependent selection, here implemented by varying the death rate (*γ*_*i*_) of the co-infected cells. For both plots, standard parameter values are: *ρ*_0_ = 1, *ρ*_*A*_ = *ρ*_*B*_ = *ρ*_*AB*_ = 0.9, *γ*_*i*_ = 0.1, *β*_*A*_ = *β*_*B*_ = 0.2, *β*_*AB*_ = 0, *m*_*i*_ = 1/3, *q*_*i*_ = 1/2, *s*_*i*_ = 1/1000 *k*_*A*,*B*_ = *k*_*B*,*A*_ = 1/2, *k*_*A*,*AB*_ = *k*_*B*,*AB*_ = 1/4.

To explore the impact of co-infection on evolutionary outcomes, we again consider two ecologically indistinguishable plasmid variants. In a deterministic simulation, such indistinguishable competitors simply remain at their initial frequencies (figure 3*a*). However, varying certain co-infection parameters (specifically, *ρ*_*AB*_, *γ*_*AB*_, *q*_*i*_, *β*_*AB*_, *g*_*i*_ or the ratio between *k*_*i*,*j*_ and *k*_*i*,*AB*_), while keeping all other parameter values identical for the two plasmid variants, changes plasmid dynamics by introducing frequency-dependent selection. This link between specific co-infection parameters and frequency-dependent selection is derived in the electronic supplementary material, text 3 and verified by simulation (electronic supplementary material, figures S2 and S3). The general insight (figure 3*a*) is that frequency dependence arises from the impact of co-infection on the plasmid variants: when co-infection is beneficial for both co-residents, we observe negative frequency-dependent selection (NFDS); when it is detrimental to both variants, we observe positive frequency-dependent selection (PFDS).

This frequency-dependence arises because the frequency of a plasmid variant determines the relative contribution of the co-infected state to its overall reproductive success, which depends on both *P*_*A*_ (*P*_*B*_) and *P*_*AB*_. If variant A is rarer than variant B (*P*_*A*_ < *P*_*B*_), the co-infected state makes up a larger proportion of the overall density of plasmid A (*P*_*AB*_/(*P*_*AB*_ + *P*_*A*_) > *P*_*AB*_/(*P*_*AB*_ + *P*_*B*_)). Therefore, if the co-infected state is beneficial for both plasmids, rare variants have an advantage, which will equalize variant frequencies. Conversely, if the co-infected state is detrimental, rare variants have a disadvantage, allowing the variant with a higher initial frequency to exclude the other. Intuitively, the co-infected state is beneficial when co-infected cells have a higher net growth rate; an overall higher conjugation rate; a lower probability of complete segregation loss; or are less susceptible to further infection with other plasmids (electronic supplementary material, text 3).

Next, we explore the effect of introducing a fitness difference between the plasmids (figure 3*b*). As expected, both NFDS and PFDS can lead to persistence of the lower fitness variant: NFDS by allowing co-existence of the two competitors, and PFDS by preventing the higher fitness variant from invading a population in which the lower fitness variant is already established. In both cases, whether the lower fitness variant is maintained depends on the strength of the frequency-dependent selection relative to the fitness difference. The frequency-dependent effect is stronger when co-infection is common. Thus, parameters which do not themselves introduce frequency-dependent selection but affect the frequency of the co-infected state (e.g. *m*_*i*_ and *k*_*i*,*j*_), can influence evolutionary outcomes by modulating the strength of frequency-dependent effects.

Finally, we consider the impact of asymmetric co-infection related effects. Thus far, we analysed effects which are equally beneficial or detrimental for both co-infecting variants: either because they impact properties of the host cell (e.g. *ρ*_*AB*_), or because we have assumed within-host interactions to be symmetric (e.g. *q*_*A*_ = *q*_*B*_, *m*_*A*_ = *m*_*B*_, …). However, within-host interactions can also be asymmetric (see §3): for example, between incompatible plasmids, an advantage in replication and/or partitioning would translate to a difference in partial segregation loss (*m*_*i*_ < *m*_*j*_) and conjugation from co-infected cells (*q*_*i*_ > *q*_*j*_) through changes in within-cell variant frequencies. Such asymmetric effects give one of the variants a competitive advantage (electronic supplementary material, figure S4), but do not, in themselves, introduce frequency-dependent effects (electronic supplementary material, text 1.4).

## 5. Discussion

This work provides an overview of the biological processes relevant in plasmid co-infection (§3) and discusses how they can be captured appropriately in a modelling framework (§2). We demonstrate how this general framework can be applied to understand how co-infection parameters shape plasmid variant selection and diversity (§4).

One insight from the modelling developed here is that co-infection can give rise to frequency-dependent selection on plasmid variants. This insight allows predictions about the evolutionary and ecological dynamics of plasmid traits. When co-infection is beneficial for the plasmid variants, this frequency-dependence is negative, which acts to maintain diversity. Beneficial interactions between co-infecting plasmids would arise, for example, from ‘collaborative’ (i.e. higher overall) conjugation from co-infected cells; positive epistasis in host fitness (reduced plasmid cost or higher plasmid benefit); or distinct *cis*-acting exclusion systems (protecting the cell from further infection with either variant). Conversely, when co-infection is detrimental, the frequency-dependence is positive, which gives high frequency variants an advantage and thus makes displacement of established variants difficult. Detrimental interactions would arise, for example, from negative epistasis or the presence of addiction systems. Finally, replication or partitioning incompatibility does not in itself lead to frequency-dependent selection. Instead, it modulates the strength of frequency-dependence that arises from other factors by decreasing the density of co-infected cells.

It is interesting to draw a parallel between these frequency-dependent effects and the concepts of over- and underdominance in population genetics. For a single locus with two possible alleles in a diploid population, heterozygotes having a fitness advantage over either homozygote leads to NFDS and maintains coexistence of the two alleles (overdominance), while heterozygotes having a fitness disadvantage leads to PFDS and drives one allele to fixation (underdominance). Over- and underdominance arise from effects on the reproductive success of individuals—i.e. in our vocabulary, effects on vertical transmission. Our results show that, in the plasmid context, this type of frequency-dependence can arise from effects on either vertical or horizontal transmission.

In addition to these general insights, the modelling framework presented here is a starting point for exploring more specific questions about the evolution and diversity of plasmid traits. For example, using empirically determined parameter ranges, this model can be used to elucidate the relative importance of different processes in maintaining the diversity of specific plasmids or plasmid traits. Furthermore, the model presented here can be extended to study more complex systems, including multiple plasmid variants, and the evolutionary stability of particular variants. Such approaches will allow determination of the role that the frequency-dependent effects described here play in shaping observed plasmid diversity.

Effects relating to plasmid co-infection also have implications on the evolutionary trajectories of bacterial populations more broadly. First, co-infection influences the rate at which bacterial populations acquire new genes through plasmid transfer: the entry of plasmids from other bacterial cells or species is influenced by the presence of a resident plasmid [7,17]. In particular, for multi-copy plasmids, the fixation of beneficial genes can be slow: the low rate of segregation loss means it may take many generations to eliminate plasmid variants without the beneficial gene—even under strong selection pressure [88]. Furthermore, the frequency-dependent effects we describe will affect the rate at which bacterial population can acquire new genes via plasmids: by promoting the introduction of new variants, negative frequency dependence will act to increase the acquisition of plasmids from other bacterial populations/species. Conversely, positive frequency dependence will act as a barrier to new plasmids entering the population, thus slowing this acquisition.

Second, co-infection also governs the extent of plasmid gene sharing. When present in the same cell, plasmids can exchange genetic material through for example, recombination [68,89]. Frequency-dependent effects would also be expected to influence the mobility of genes between plasmids (or plasmid and chromosome [43]). For example, if the presence of the same cargo gene on co-resident plasmids gives rise to negative epistasis between the plasmids (owing to negative gene dosage effects), the resulting PFDS would constrain gene mobility: the disadvantage associated with low frequency variants would prevent plasmids that have newly acquired the cargo gene from increasing in frequency.

Our results are closely linked to previous theoretical work on epidemiological models of co-infection [36], which has highlighted how model structure can include coexistence-promoting mechanisms. Specifically, the motivating concern of this previous work was that models of co-infection typically implicitly and unintentionally assumed that a host carrying one strain would be susceptible to co-infection with another strain, but protected from re-infection with itself: co-infection was possible, but displacement was neglected. This is akin to assuming *cis*-acting exclusion. In models of plasmid co-infection, this specific concern is—to some extent—less acute, as *cis*-acting exclusion systems are thought to be widespread among conjugative plasmids [7]. If these systems are indeed as effective *in vivo* as *in vitro* data suggest, co-infection only occurs between plasmids of different exclusion groups and co-infected cells are therefore indeed not susceptible to displacement. Furthermore, when considering variants of the same backbone with and without a particular cargo gene, it is appropriate to exclude co-infection [11,43]. On the other hand, our results highlight that frequency-dependent effects also arise from other model features. Many of these effects are linked to copy number, making evolutionary outcomes heavily dependent on how co-infecting plasmids influence each others’ copy numbers. It is thus important to be explicit about the traits of the modelled variants and aware that results may not generalize for different assumptions about plasmid backbones.

A key feature of the framework discussed here is that cells are tracked in terms of the plasmid variants they carry, without explicitly incorporating plasmid copy number: each cell type (*P*_0_, *P*_*A*_, *P*_*B*_, *P*_*AB*_) is represented in terms of the average cell, and heterogeneity within cell types is ignored. This is a standard approximation in compartmental models, but warrants additional discussion in the context of co-infection. First, this approximation can make the link between model and biological processes less intuitive and complicates parametrization, as processes which change within-cell plasmid frequencies have to be represented in terms of average plasmid loss. Second, by representing the co-infected state as a single variable, the average frequency of plasmid variants within co-infected cells is implicitly specified. This highlights the importance of carefully considering how certain parameters values depend on relative plasmid frequencies (e.g. *k*, *m*, *q*), particularly when modelling plasmids where one variant has a within-cell competitive advantage and thus the variant frequencies within-co-infected cells are not equal. Overall, the contexts in which explicit models of plasmid copy number are not satisfactorily approximated by average copy numbers warrants further exploration (electronic supplementary material, text 2.2).

While experimental studies have provided—and continue to provide—central insights into plasmid co-infection and its ecological and evolutionary implications, a full understanding of these implications also requires more data on the natural occurrence and distribution of plasmid co-infection. This includes population-level studies investigating the prevalence of plasmid co-infection across bacterial phyla (expanding on e.g. [17,18]), as well as its correlation with incompatibility group, plasmid size and copy number. Furthermore, while studied in detail at the mechanistic level, little is known about the natural diversity and phenotypic effects of various exclusion and TA systems. Carefully designed bioinformatics studies could address some of these questions. However, sequencing databases are currently not representative of natural microbial diversity, and the meta-data to account for phylogenetic, geospatial or phenotypic biases is often lacking [90]. Additionally, plasmids may not be represented accurately in the deposited genomes [91,92], complicating conclusions on overall plasmid co-infection.

A combination of empirical and theoretical approaches is necessary to iteratively refine our understanding of plasmid diversity: on the one hand, using empirical data to inform model parameter values and processes, and on the other, evaluating the results of simulations against natural and experimental observations. In particular, combining insights into the mechanistic effects of specific traits from experimental studies and data on the distribution of these traits in natural plasmid populations is a crucial step in gaining predictive understanding of plasmid co-infection. Modelling can provide an important tool in bridging these two levels of observation, for example by providing testable predictions for controlled laboratory microcosm studies with multiple conjugative plasmids and identifying determining (mechanistic) factors causing mismatch with empirical (population-level) data. Through careful consideration of the biological processes and potential modelling pitfalls relating to plasmid co-infection, we have developed a modelling framework which can serve as a basis for such future work.

## Data accessibility

This article has no additional data.
